# Isolation and Characterization of Potential Starter Cultures from the Nigerian Fermented Milk Product *nono*

**DOI:** 10.3390/microorganisms9030640

**Published:** 2021-03-19

**Authors:** Olakunle Fagbemigun, Gyu-Sung Cho, Niels Rösch, Erik Brinks, Katrin Schrader, Wilhelm Bockelmann, Folarin A. Oguntoyinbo, Charles M. A. P. Franz

**Affiliations:** 1 Department of Microbiology, Faculty of Science, University of Lagos, Akoka, 100213 Lagos, Nigeria; kunlefagbemigun@gmail.com; 2 Department of Microbiology and Biotechnology, Max Rubner-Institut, Hermann-Weigmann-Straße 1, 24103 Kiel, Germany; gyusung.cho@mri.bund.de (G.-S.C.); Niels.Roesch@mri.bund.de (N.R.); Erik.Brinks@mri.bund.de (E.B.); Wilhelm.Bockelmann@mri.bund.de (W.B.); Charles.Franz@mri.bund.de (C.M.A.P.F.); 3 Department of Safety and Quality of Milk and Fish Products, Max Rubner-Institut, Hermann-Weigmann-Straße 1, 24103 Kiel, Germany; Katrin.Schrader@mri.bund.de; 4 A.R. Smith Department of Chemistry and Fermentation Sciences, Appalachian State University, 730 River Street, Boone, NC 28608, USA

**Keywords:** *nono*, genome, milk, lactic acid bacteria, fermentation

## Abstract

*Nono*, an important traditional fermented dairy food produced from cow’s milk in Nigeria, was studied for microbial diversity and for starter culture development for industrial production. On the basis of a polyphasic approach, including phenotypic and genotypic methods such as 16S rRNA gene sequencing, repetitive element PCR (rep-PCR) fingerprinting metagenomics, and whole genome sequencing, we identified *Lactobacillus* (*Lb.*) *helveticus*, *Limosilactobacillus (L.) fermentum*, *Lb. delbrueckii*, and *Streptococcus* (*S.*) *thermophilus* as predominant bacterial species involved with milk fermentation during traditional *nono* production in Nigeria, while the predominant yeast species in *nono* was identified as *Saccharomyces cerevisiae*. Using metagenomics, *Shigella* and potential pathogens such as enterobacteria were detected at low levels of abundance. Strains of the predominant lactic acid bacteria (LAB) were selected for starter cultures combination on the basis of their capacities for rapid growth in milk and reduction of pH below 4.5 and their gelling characteristic, which was demonstrated noticeably only by the *S. thermophilus* strains. Whole genome sequence analysis of selected bacterial strains showed the largest assembled genome size to be 2,169,635 bp in *Lb. helveticus* 314, while the smallest genome size was 1,785,639 bp in *Lb. delbrueckii* 328M. Genes encoding bacteriocins were not detected in all the strains, but all the LAB possessed genes potentially involved in diacetyl production and citrate metabolism. These bacteria isolated from *nono* can thus be used to improve the microbial safety quality of *nono* in Nigeria, in addition to improving technological parameters such as gelling viscosity, palatability, and product consistency.

## 1. Introduction

*Nono* is a spontaneously lactic acid fermented milk that originates from the northern parts of Nigeria. It is predominantly prepared and sold in local markets by the Hausa/Fulani cattle herdsmen [1,2]. Generally, *nono* is prepared in homes from unpasteurised cow’s milk collected in a calabash and allowed to ferment naturally for 24 h [3,4,5] before being sold to rural and urban people on markets [6]. Depending on uncontrolled production processes and too poor hygiene during preparation, bacteria other than lactic acid bacteria (LAB), including foodborne pathogenic bacteria such as *Escherichia coli*, *Salmonella, Shigella, Staphylococcus aureus*, and *Bacillus cereus* can occur in these products [2,5,6,7,8]. The presence of foodborne pathogens in such products suggests that the *nono* products are probably not fermented to a pH sufficiently fast and low enough to inhibit such bacteria. Furthermore, the production of *nono* by spontaneous fermentation with unpasteurized milk leads to varying products with different levels of quality inconsistency and safety. In order to obtain a well-controlled nono fermentation with a fast and deep acidification, one would benefit from the use of starter cultures. Upon inoculation of pasteurized or non-pasteurized milk with suitable starters at high concentration, the pH could be rapidly lowered to pH 4.50–4.0, at which many foodborne pathogens will not grow [9].

Thus far, there have not been many studies on the diversity of LAB found during fermentation of *nono.* Banwo et al. [10] showed that quite a diverse range of LAB were associated with *nono* production, identifying *Lactobacillus* (*Lb.*) *plantarum*, *Enterococcus* spp., and *Pediococcus* spp., while Okagbue and Bankole [1] identified *Lactococcus* (*Lc.*) *lactis* subsp. *lactis* biovar diacetylactis (in the original publication termed *Streptococcus diacetilactis*), *Lb. brevis*, and *Saccharomyces* (*S.) cerevisiae* to be associated with the fermentation. The authors of [11] isolated *Lb. plantarum, Lb. brevis, Lb. delbrueckii* subsp. *bulgaricus*, and *Limosilactobacillus fermentum* from *nono*. In addition, the authors [11] indicated that yeasts, especially *S. cerevisiae*, were also associated with the fermentation. The aim of this study was, therefore, to characterize the predominant LAB and yeast species associated with *nono* fermentation and to perform a preliminary investigation of their technological properties in order to select potential starter cultures for controlled *nono* production.

## 2. Materials and Methods

### 2.1. Microbiological Analysis of Nono Samples from the Nigerian Market

For a general microbiological analysis, 25 *nono* samples were bought from Fulani processors and local markets in Kano, Katsina, Jigawa, and Bauchi states in northern Nigeria. The samples were transported under cooled conditions to the laboratory, and the pH of the *nono* samples was measured using a pH electrode (Mettler Tuledo Inlab Expert Pt 1000, Gießen, Germany). Samples were then diluted in a serial tenfold-dilution series using quarter-strength Ringers solution (Merck, Darmstadt, Germany). After this, 100 µL aliquots of appropriate dilutions were plated out in duplicate onto different selective culture media in order to determine the total amount of aerobic, mesophilic bacteria on plate count agar (VWR Chemicals, Leuven, Belgium); LAB on de Man, Rogosa, and Sharpe agar (MRS agar, Merck, Darmstadt, Germany); and Gram-negative bacteria on violet red bile dextrose agar (VRBD agar, Merck, Darmstadt, Germany) incubated at 30 °C and streptococci on M17 medium incubated at 45 °C. In addition to selective enumeration of bacteria, the numbers of yeasts in the samples were determined using yeast extract–glucose–chloramphenicol agar (YGC agar, Merck, Darmstadt, Germany).

### 2.2. S Amplicon Sequencing Analysis of the Nono Bacterial Community

The bacterial community composition of the *nono* samples was also determined using tag-encoded, 16S rRNA gene MiSeq-based (Illumina, San Diego, CA, USA) high-throughput sequencing. The total bacterial DNA was extracted using the repeated bead beating plus column (RBB+C) method as described previously by Yu and Morrison [12] from 20 mL of each of the products. The DNA concentration of the samples was measured using the Qubit 3.0 fluorometer with the high sensitivity kit (Thermo Scientific, Darmstadt, Germany) and was then diluted to 5 ng/µL. DNA amplification was carried out following the two-stage PCR protocol provided by Illumina. The first PCR step was carried out in a final volume of 25 µL containing 2.5 µL template DNA (5 ng/µL), 5 µL of the primers 16S Fw (5′-TCG TCG GCA GCG TCA GAT GTG TAT AAG AGA CAG CCT ACG GGN GGC WGC AG-3′) and 16S Rev (5′-GTC TCG TGG GCT CGG AGA TGT GTA TAA GAG ACA GGA CTA CHV GGG TAT CTA ATC C-3′) binding to the the V3 and V4 hypervariable regions of the 16S rRNA gene, respectively (final concentration 1 µM) (Illumina 16S Metagenomic Sequencing Library Preparation protocol), and 12.5 µL of KAPA HiFi HotStart Ready Mix (Kapa Biosystems, Inc. Wilmington, MA, USA). The PCR reactions were performed in a PeqSTAR thermal cycler (Peqlab, Erlangen, Germany) using the following program: initial denaturation at 95 °C for 3 min, followed by 25 cycles of 95 °C for 30 s, 55 °C for 30 s, and 72 °C for 30 s, with a final elongation at 72 °C for 5 min. The sizes of the 16S rRNA gene PCR products were confirmed by automated electrophoresis using the Experion Automated Electrophoresis System (BioRad, Munich, Germany). The PCR products were cleaned using AMPure XP magnetic beads (Agencourt) and then used as a template for the second PCR, the index PCR, to attach dual indices with the Nextera XT Index kit (Illumina). PCR was performed using 25 µL KAPA HiFi HotStart Ready Mix (KAPPA BIOSYSTEMS), with an initial denaturation at 95 °C for 3 min, followed by 8 amplification cycles (denaturing at 95 °C for 30 s; annealing at 55 °C for 30 s; extension at 72 °C for 30 s) with a final extension at 72 °C for 5 min. The index PCR products were cleaned with AMPure XP beads (Agencourt) and the final DNA concentration was measured using the Qubit 3.0 fluorometer with the broad range kit (Thermo Scientific). The final DNA concentration of each sample was adjusted to 4 nM with 10 mM Tris (pH 8.5), and 5 µL of diluted DNA from each sample were mixed together for pooling the libraries with unique indices. The pooled samples were further diluted using the Illumina Hybridization Buffer (HT1) to a final loading concentration of 6 pM. Sequencing of the 16S rRNA gene-based metagenome was performed in Miseq Illumina following the Illumina instructions for metagenomics workflows. The resulting fastq files were analyzed using the *de novo* analysis pipeline at the IMNGS platform for ecology and diversity studies [13]. The demultiplexed Illumina fastq files were pre-processed using the remultiplexer pearl script recommended by the IMNGS platform and unknown sequences belonging to ambiguous sequences, archaea, eukaryota, chloroplasts, and mitochondria were excluded. The processed sequences were uploaded to the MG-RAST and analyzed using the RDP taxonomic database. The relative abundance of OTUs were calculated and visualized using the implanted pipelines of MG-RAST.

### 2.3. Isolation and Identification of Predominant Lactic Acid Bacteria and Yeasts from Nono

In order to accurately characterize the predominant lactic acid bacteria and yeasts associated with *nono* fermentation, we isolated colonies from MRS agar and YGC agar plates used to determine the lactic acid bacterial and yeast counts, respectively. For the LAB identification, a polyphasic approach to identify the strains was adopted. For this, strains were first presumptively identified as LAB by phenotypic tests and then grouped by repetitive element PCR (rep-PCR) fingerprinting to the species level [14,15]. Representative isolates of the major groups were then characterized by 16S rRNA gene sequencing, indicating the rest of the closely grouped isolates to presumptively be the same species. Of these isolates, the genomes of selected strains with potential for use as starter culture were sequenced in order to determine the presence of technologically important genes for the fermentation. Colonies were randomly selected from the plates onto which the highest dilutions were plated. For LAB, colonies were purified by streaking onto MRS agar plates (at least 3 times). A total of 223 presumptive lactic acid bacteria strains thus isolated were tested for Gram-reaction using the 3% KOH method, for the presence of the enzyme catalase, and for production of CO_2_ from glucose fermentation, as described previously [14,15]. Of these, 147 were selected for further characterization by rep-PCR.

Yeasts were purified by streaking onto YGC agar, cultured on malt extract agar (2% malt extract, Carl Roth; 0.1% peptone from soybean, Fluka; 1.5% agar-agar, VWR) at 25 °C. A total of 43 isolates were identified using a commercial test kit (API BioMérieux ID 32 C, Nürtingen, Germany), which tests for utilization of different sugars, and the results were transformed into an API code that is analyzed using the database of the apiweb (BioMérieux) and the Westerdijk Fungal biodiversity institute.

The total genomic DNA was isolated from each of the 147 presumptive LAB strains according to the method of [16] as modified by [17], and the bacteria were fingerprinted using rep-PCR fingerprinting as previously described [14]. Rep-PCR was performed using the primer GTG^5^ (5′-GTG GTG GTG GTG GTG-3′), and PCR products were separated by electrophoresis on a 1.8% (w/v) agarose gel using 1 × TBE buffer. Groupings of rep-PCR fingerprints were performed by means of the Pearson product-moment correlation coefficient (*r*) and the unweighted pair-group method using arithmetic averages clustering algorithm (UPGMA) [18] using the Bionumerics (version 7.2.6) software package (Applied Maths, Sint-Martens-Latem, Belgium).

The almost complete 16S rRNA gene (maximal 1540 bp) was also amplified by PCR from total genomic DNA extracted as described above from 16 isolates characteristic of major rep-PCR groups. The PCR products were amplified in 50 µL volumes containing 100 ng template DNA, 1 × Taq DNA polymerase buffer (Genaxxon, Ulm, Germany), 125 µM of each dNTP (CarlRoth, Karlsruhe, Germany), 25 pM of each forward and reverse primer (16S fw 5′-AGA GTT TGA TCM TGG CTC AG-3′ and 16S rev 5′-TAC GGY TAC CTT GTT ACG ACT-3′), and 1.5 U Taq DNA polymerase (Genaxxon, Ulm, Germany). The PCR reaction was performed using an initial denaturation step at 94 °C for 3 min, followed by 32 cycles of 94 °C for 30 s, 55 °C primer annealing for 30 s, 72 °C extension for 1 min 30 s, followed by a final extension step at 72 °C for 5 min. All PCR products were purified using PCR cleaning columns (Peqlab) and subsequently sequenced at Eurofins (Eruofin, Cologne, Germany). The sequences of 16S rRNA gene PCR products were compared to those present in the EzTaxon database [19].

The total genomic DNA of each of the selected potential starter cultures was isolated using the peqGOLD bacterial DNA kit (Peqlab, Erlangen, Germany) after growing the strain overnight at 30 °C in MRS broth. The sequencing library was constructed using the Nextera Flex DNA library kit (Illumina, San Diego, CA, USA) and sequenced with 2 × 251 bp reads on an Illumina MiSeq platform. The Trimmomatic version 0.36 for trimming of Illumina paired-end [20] software was used to remove adapter sequences with the following parameters (sliding window; 4:15 and leading;3) as well as low-quality reads, and reads with a minimum contig length of 500 bp and fivefold coverage were then *de novo* assembled using SPAdes v3.13.1 with careful and k-mer 77 parameters [21]. Annotation of the draft genome sequences was performed with the NCBI Prokaryotic Genome Annotation Pipeline v4.10 using default parameters [22]. The whole genome sequences of the *Lb. helveticus* strain 314 and *Streptococcus* (*S.*) *thermophilus* strain 252 have been deposited in DDB/GenBank under the accession numbers JAFIWI000000000 and JAFIWH000000000.

### 2.4. Milk Fermentation with Starter Lactic Acid Bacteria and Yeast Strains

To determine the growth and acidification ability of potential starter strains, we obtained pasteurized cow’s milk used in fermentation experiments from the Max Rubner-Institute’s experimental farm located in Schädtbek just outside the city of Kiel. The acidifying capacity of selected strains (*S. thermophilus* strains 252, 349, and 363; *Lb. helveticus* strains 058, 314, and 321; *L. fermentum* strains 054, 317 and 339; as well as *Lb. delbrueckii* strains 052, 053, and 328M) was tested in freshly pasteurized (90–95 °C for 15 min) whole milk at 30 °C for 24 h. Strains from these 4 species were chosen, as they represent the predominant LAB species associated with *nono* samples (see below). An incubation temperature of 30 °C was chosen as *nono* fermentation was performed according to traditional local methods, where the milk is left to ferment at ambient temperatures that can reach 30 °C in Nigeria. For the acidification experiments, the strains were inoculated at 1 × 10^6^ cfu/mL in single-strain fermentations. The milk pH was measured after 2, 4, 6, and 24 h fermentation. The growth of the potential starter strains was assessed upon inoculation of the milk at 0 and after 24 h fermentation by plating 100 µL of appropriate tenfold dilutions of the milk in quarter-strength Ringers solution onto MRS agar, and incubating plates for 48 h at 30 °C under gas pack anaerobically. 

On the basis of pH reducing capacity and growth ability in the single-strain fermentation tests, we chose the *Lb. helveticus* 314, *L. fermentum* 317 [23], *S. thermophilus* 252, *Lb. delbrueckii* 328M, and the yeast *S. cerevisiae* 370 strains as possible starter cultures to test *nono* production at laboratory scale in 50 mL volumes. These strains were tested as multi-strain starters for fermentation in co-cultures and included all 4 bacterial strains inoculated to a level of 1 × 10^6^ cfu/mL and the yeast inoculated at 1 × 10^4^ cfu/mL (starter A), as well as the 4 bacterial strains-inoculated at a level of 1 × 10^6^ cfu/mL without the yeast (starter B). The yeast was chosen because Okagbue and Bankole [1] reported that *S. cerevisiae* was consistently isolated from *nono*.

The milk was fermented in 50 mL volumes at 30 °C for 48 h, and the pH and numbers of bacteria were determined after 0 h (immediately after inoculation), 24 h, and 48 h. For enumeration, 1 mL of milk was collected and diluted in quarter-strength Ringer’s solution in a tenfold dilution series. Hundred microliter aliquots from appropriate dilutions were plated out onto MRS agar plates for determining the *Lactobacillus* sp. counts and on M17 agar for the *S. thermophilus* counts (only for the fermentation that contained *S. thermophilus* 252). The yeast counts were determined by plating onto YGC agar, to which 0.2% of a 10% tartaric acid solution was added after autoclaving in order to adjust the pH to 4.6. Both MRS agar (anaerobic) and M17 agar plates (aerobic) were incubated at 30 °C for 48 to 72 h, while YGC agar plates were incubated at 25 °C for 72 h.

### 2.5. Determination of Rheological Properties

For the measurement of the viscosity, a rheometer MCR 302 (Anton Paar, Ostfildern, Germany) was used. Aliquots of 0.7 mL of the fermented product were placed into the cone and plate measuring system (diameter 50 mm) using a syringe without needle. A flow curve was recorded at a temperature of 20 °C using a logarithmic ramp of the shear rate (10 to 1000 s^−1^). These measurements were performed after 24 h and 48 h of fermentation.

## 3. Results

### 3.1. Microbiological Analysis of Nono Samples from the Nigerian Market

**Figure 1:**
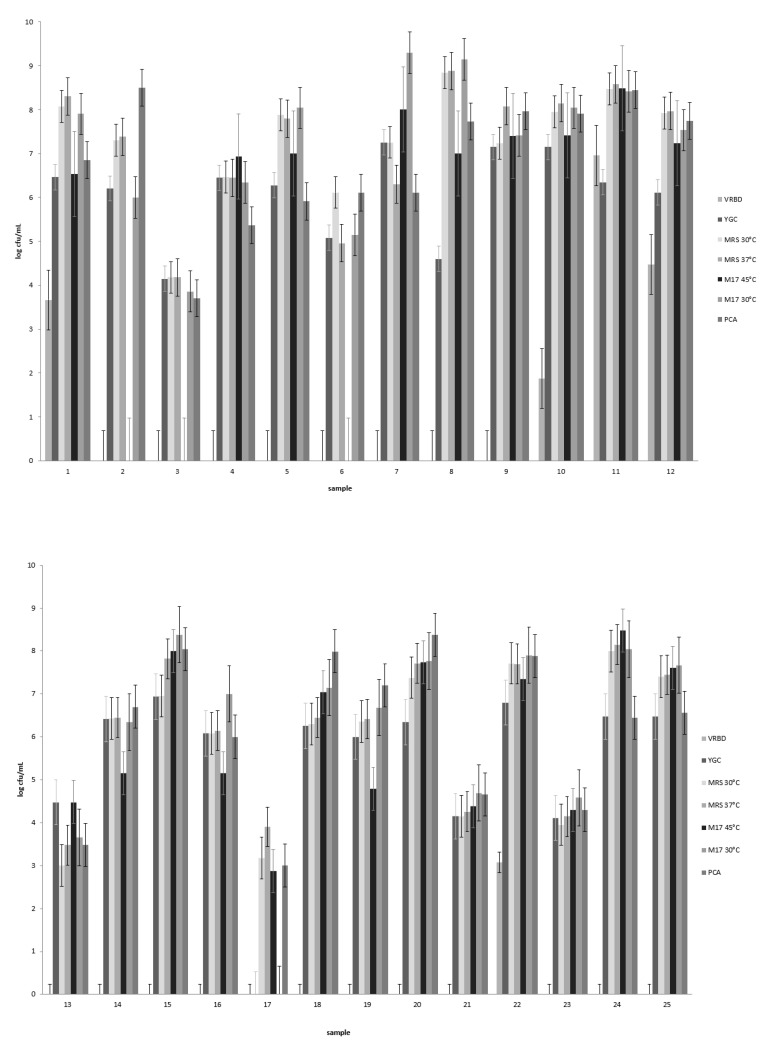
Bacterial counts in 25 *nono* samples obtained from local Fulani processors and markets in Kano, Katsina, Jigawa, and Bauchi states in northern Nigeria. Enterobacteria counts were determined on violet red bile agar (VRBD), yeasts on yeast extract–glucose–chloramphenicol agar (YGC); lactic acid bacteria on de Man, Rogosa, and Sharpe medium (MRS); and lactococci and streptococci on M17 medium. The total aerobic, mesophilic counts were determined on plate count agar (PCA), and the standard error is shown. See text for details of incubation conditions.

*Nono* samples obtained from various Nigerian markets showed a large variation in bacterial counts (Figure 1). Gram-negative bacteria were detected in 5 of the 25 samples at levels ranging from 10^2^ to 10^7^ cfu/mL. Lactic acid bacterial counts determined at 30 °C ranged from 10^3^ to 10^9^ cfu/mL, with 20 of 25 samples showing counts of 10^6^ cfu/mL or higher. Generally, the LAB counts determined at 37 °C were very similar and differed by only 0.5 cfu/mL (Figure 1). The numbers of presumptive streptococci were determined on M17 agar at 45 °C, and streptococci were present in 22 of the 25 *nono* samples. In these, streptococci numbers ranged from ≈10^3^ to 10^8^ cfu/mL, with counts being higher than 10^6^ in 14 of the *nono* samples. Yeasts occurred in 24 out of 25 *nono* samples and their counts ranged from 10^4^ to 10^7^ (Figure 1), and 18 samples showed counts higher than 10^6^ cfu/mL. Total aerobic, mesophilic counts ranged from 10^3^ to 10^8^ cfu/mL (Figure 1).

### 3.2. Metagenomic Analysis

**Figure 2:**
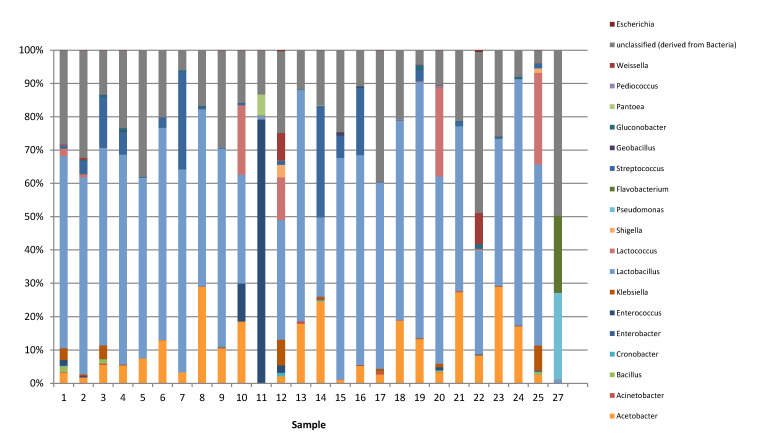
Relative abundance of microorganisms in 25 *nono* samples obtained from local Fulani processors and markets in Kano, Katsina, Jigawa, and Bauchi states in northern Nigeria, as determined by 16S-based amplicon sequencing.

The 16S rRNA amplicon sequencing was used for the metagenomic study with primers targeting the V3/V4 region of the 16S rRNA gene that is specific for different genera of bacteria. The results of the metagenomics-based biodiversity study are shown in Figure 2. In total, 5,059,742 numbers of OTUs were analyzed in this study, and the average number of OTUs was 202,389. After clustering all OTUs using the RDP taxonomic database, bacteria of the genus *Lactobacillus* appeared to occur in all samples (Figure 2) up to 76% of relative sequence abundance, depending on the sample. Four samples (2, 5, 17, and 22) contained over 30% OTUs as unclassified sequences. Bacteria of the genus *Lactococcus* and *Streptococcus* were also identified as predominantly occurring lactic acid bacteria in many of the products at a widely varying relative abundance of up to 27% and to 34% of relative sequence abundances, respectively, again depending on the sample. In most *nono* samples, lactobacilli and lactococci/streptococci were the predominant microorganisms (combined relative sequence abundances up to 89%).

### 3.3. Rep-PCR Fingerprinting of LAB and Yeast Identification

**Figure 3:**
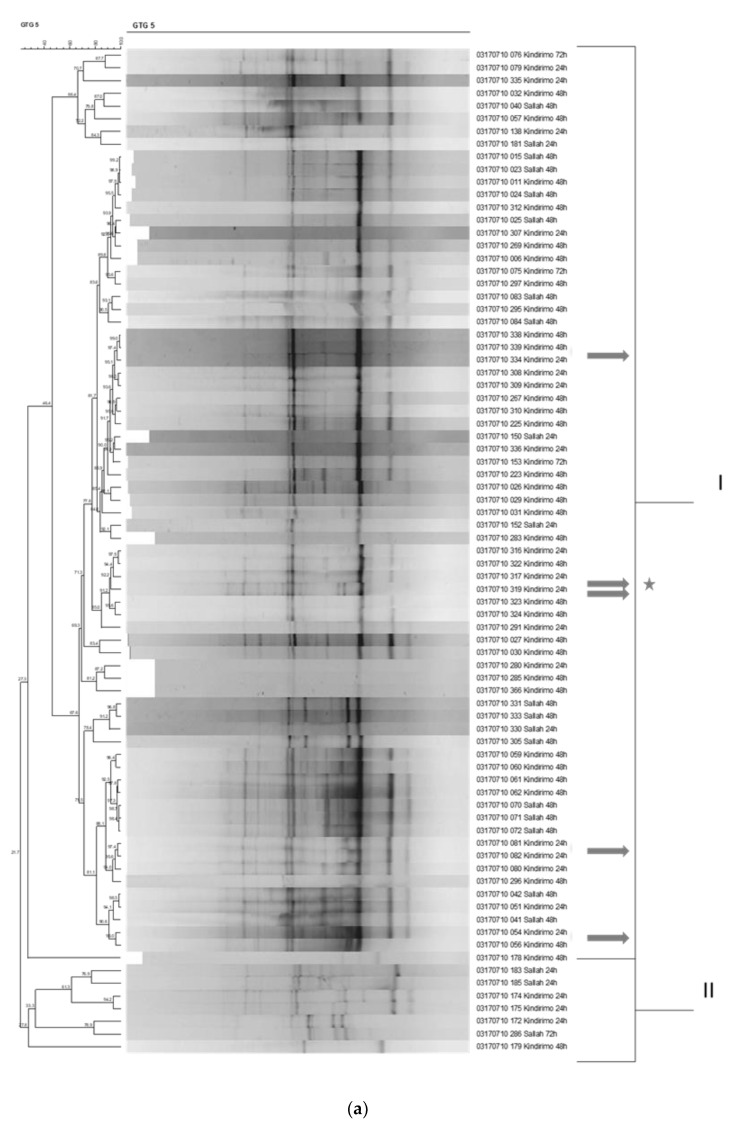

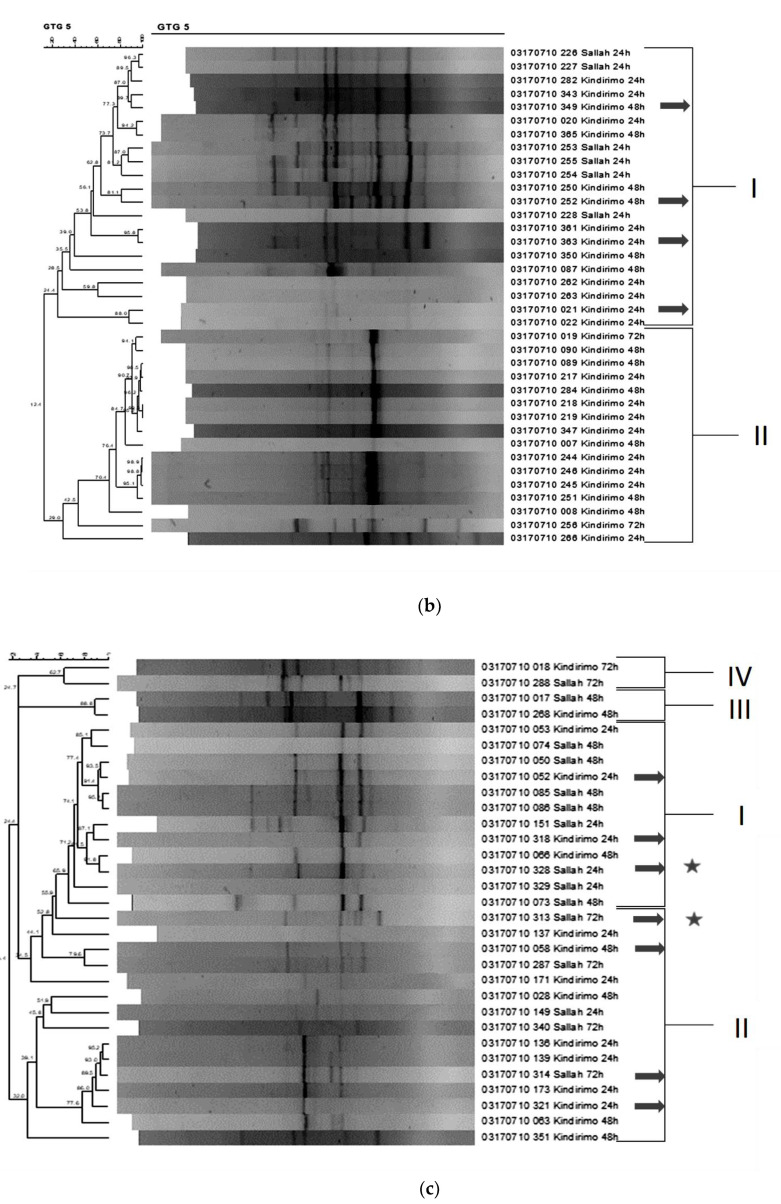
Dendrogram obtained by unweighted pair-group method using arithmetic averages clustering algorithm (UPGMA) (I-IV clusters) of correlation value *r* of repetitive element PCR (rep-PCR) fingerprint patterns with primer GTG^5^ of predominant lactic acid bacterial strains isolated from the fermented milk product *nono* from Nigeria. (**a**) Heterofermentative rod-shaped lactic acid bacteria, (**b**) homofermentative coccus-shaped lactic acid bacteria, (**c**) homofermentative rod-shaped lactic acid bacteria. Arrows indicate the strains for which the 16S rRNA gene was amplified and sequenced in order to identify. Star indicates strains *Limosilactobacillus* (*L.*) *fermentum* 317, *Lactobacillus* (*Lb.*) *delbrueckii* 328M, and *Lb. helveticus* 313 that were not selected as (potential) starter strains in this study but were sequenced in a previous study to confirm 16S rRNA gene sequencing results and to establish genome sequencing methods [23].

Repetitive element PCR (rep-PCR) was used as major fingerprinting method, and isolates were identified by sequencing the 16S rRNA gene of representative isolates selected of the de-replicated rep-PCR fingerprint clusters. The rep-PCR fingerprints of the largest group of isolates, i.e., the heterofermentative, rod-shaped bacteria, is shown in Figure 3a. Two groups could be distinguished on the basis of their fingerprint patterns. Most isolates occurred in group I The 16S rRNA genes of five representative isolates of this group (marked by an arrow) were sequenced and based on the sequence similarity to reference strains in the EzTaxon database—these isolates could be identified as *L. fermentum*, indicating that all the strains clustering in this subgroup were *L. fermentum* strains. This was confirmed by whole genome sequencing for which the strain 317 was selected to represent this predominant group, and this strain was identified as *L. fermentum* [23]. The rep-PCR fingerprinting showed that homofermentative cocci could also be grouped into two major groups on the basis of their fingerprints (Figure 3b). Most of the isolates occurred in group I, and these were identified by 16S gene sequencing as *S. thermophilus*. The bacteria occurring in group II (Figure 3b) were determined by 16S rRNA gene sequencing to consist of enterococci (results not shown). The rep-PCR fingerprints of the second largest group of isolates, the homofermentative, rod-shaped lactic acid bacteria, are shown in Figure 3c. Again, two groups could be distinguished on the basis of their fingerprint patterns, although the rep-PCR demarcation of the groups was not as stringent as for the other groups. The sequencing of 16S rRNA gene of selected strains in group I showed these to be *Lb. delbrueckii* strains, while selected strains in group II could be identified by 16S rRNA gene sequencing as *Lb. helveticus* strains (Figure 3c). This was again confirmed by whole genome sequencing in which strain 328M representative of group I and strain 313 representative of group II (Figure 3c) were chosen and could be identified as *Lb. delbrueckii* and *Lb. helveticus*, respectively [23].

Results of the yeast characterization by API ID 32C showed that the predominant yeasts occurring in the product were *S. cerevisiae* (17 of 43 strains, ≈40%) and *Pichia norvengensis* (9/43 strains, ≈21%), together comprising ≈61% of the isolates. The remainder consisted of various other yeasts such as *Klyveromyces marxianus* (3 of 43 strais, ≈7%), *Pichia kudriavzevii* (3 of 43 strains), *Kasachstania holmii* (2 of 43 strains, ≈5%), and other species (see Table A1 in Appendix A).

### 3.4. In Vitro Assessment of Growth and Acidification Abilities of Potential LAB and Yeast Starter Strains

#### 3.4.1. Single Culture Fermentations

**Figure 4:**
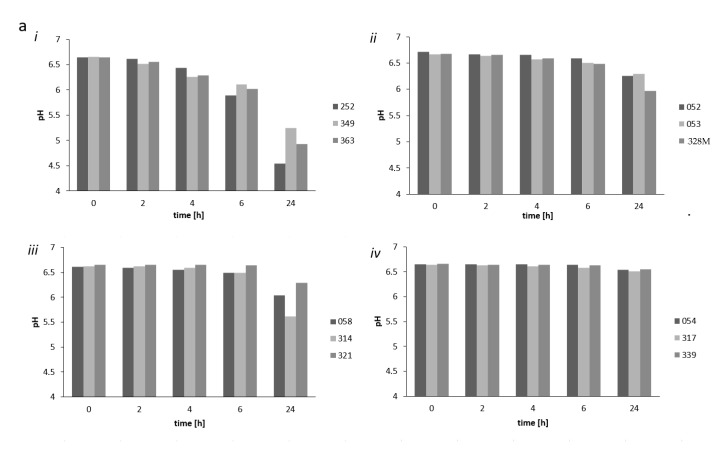

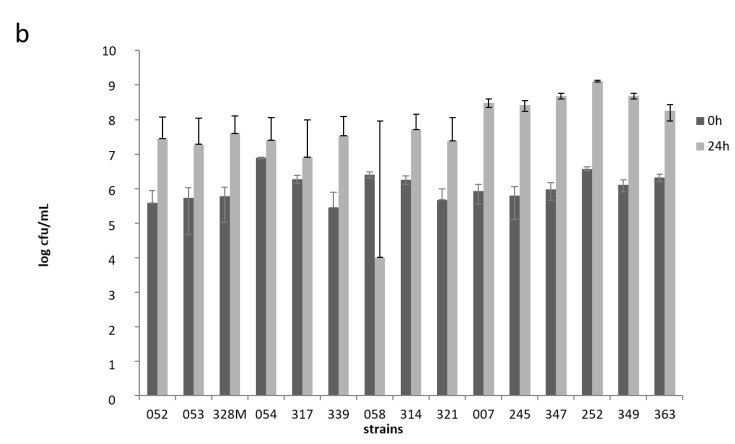
Development of pH of milk during 24 h *nono* fermentation with potential starter culture (**a**) with strains *Streptococcus* (*S.*) *thermophilus* strains 252, 363, and 349 (single determinations in a single fermentation) (i); *Lb. delbrueckii* 052, 053, and 328M (ii); *Lb. helveticus* 058, 314, and 321 (iii); and *L. fermentum* 054, 317, and 339 (iv), as well as counts (log cfu/mL) of potential *nono* starter strains in milk directly after inoculation (0 h) and after 24 h fermentation (**b**) as determined by plate counting on MRS agar (*Lb. delbrueckii*, *L. fermentum*, and *Lb. helveticus* strains) and M17 agar (*S. thermophilus* strains). The standard error in terms of duplicate counts is indicated.

Three strains each of *L. fermentum*, *Lb. helveticus*, *S. thermophilus*, and *Lb. delbrueckii* were tested for their growth and acid production in milk. The strains did not all show equal acidifying activities. Strain *Lb. helveticus* 314 showed the highest acidification capacity among the *Lb. helveticus* strains, decreasing the pH from 6.5 to 5.5 after 24 h (Figure 4a). *Streptococcus thermophilus* also had very good acidification activity, and strain 252 showed especially high acid producing capacity, decreasing the pH from 6.5 to ≈4.5 in 24 h (Figure 4a). *L. fermentum* strains did not show much acidification capacity in milk at all, while *Lb. delbrueckii* strain 328M showed the best pH reducing capacity among *Lb. delbrueckii* strains, which were generally not good acidifiers of milk, decreasing the pH from ≈6.5 to only just below pH 6.0 (Figure 4a). Most strains showed more than 1 log unit growth in milk after 24 h when inoculated as single strains, increasing from log 6.0 cfu/mL to at least log 7.0 cfu/mL (Figure 4b). The *S. thermophilus* strains 252, 349, and 363 showed especially good growth in milk increasing by 2 log unit to >1 × 10^8^ cfu/mL (Figure 4b).

#### 3.4.2. Mixed Culture Fermentations

**Figure 5:**
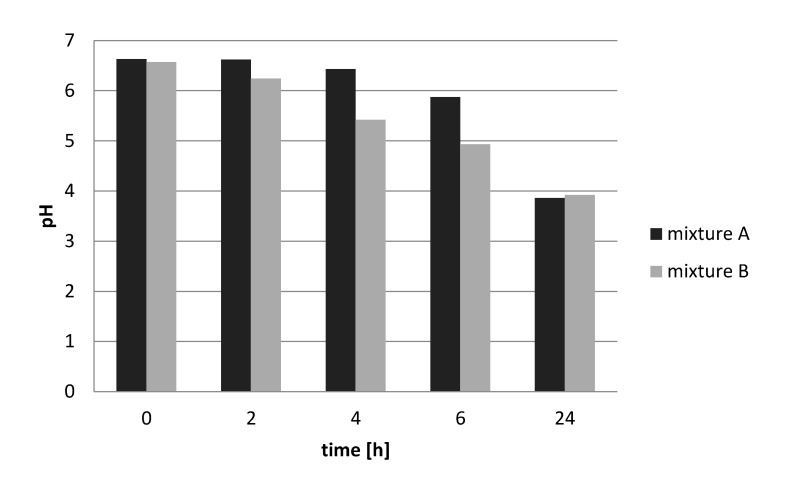
Development of pH of starter culture combinations consisting of the starter strains *Lb. helveticus* 314, *L. fermentum* 317, *S. thermophilus* 252, *Lb. delbrueckii* 328M, and the yeast *S. cerevisiae* strain 370 (mixture A) or the lactic acid bacteria strains without the yeast (mixture B) during 24 h fermentation in milk. pH determinations were done as single determinations.

Both starter culture combinations used (starter combinations A and B, i.e., with or without yeast) were equally effective in reducing the pH from ≈ 6.5 to slightly below pH 4.0 (Figure 5). The results of the mixed culture fermentations also show that *S. thermophilus* in the mixture, as determined on M17 agar, grew very well in the milk to a level of 1 × 10^9^ cfu/mL (Figure 5) in 24 h. The yeast grew from 1 × 10^4^ to 1 × 10^6^ cfu/mL over 24 h as determined on YGC agar, while the lactobacilli (*Lb. delbrueckii* and *Lb. helveticus* strains) grew on MRS agar from 1 × 10^6^/1 × 10^7^ cfu/mL (depending on the initial inoculation of the mixture) to 1 × 10^9^ cfu/mL after 24 h incubation.

### 3.5. Rheological Properties of Starter Candidates

**Figure 6:**
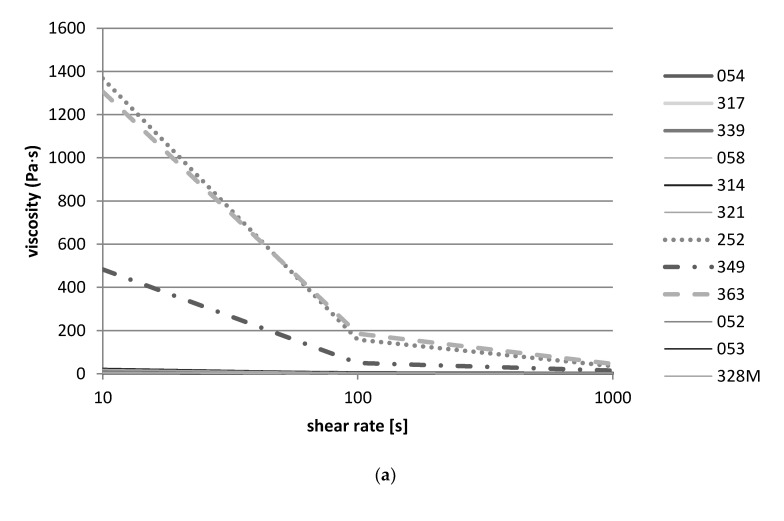

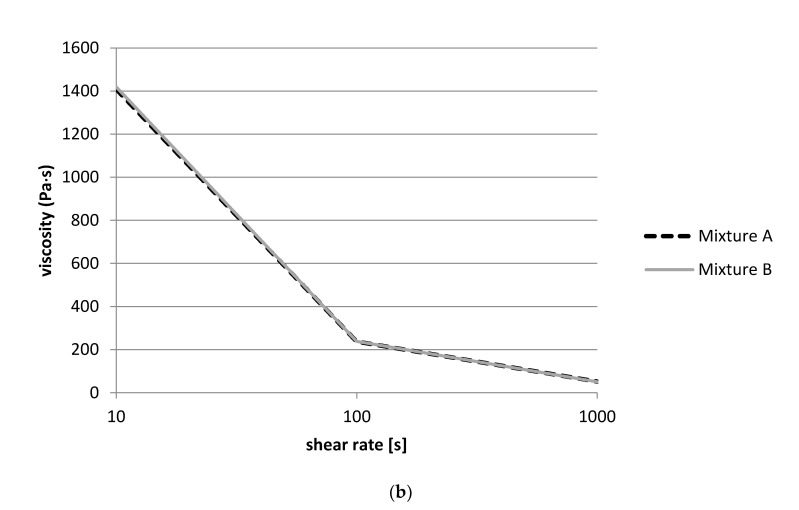
Flow curves of the milk fermented for 24 h with different single strain cultures of *Lb. delbrueckii* strains 052, 053, and 328M; *L. fermentum* strains 054, 317, and 339; *Lb. helveticus* strains 058, 314, and 321; and *S. thermophilus* strains 252, 363, and 349 (**a**), or with mixed starter strains *Lb. helveticus* 314, *L. fermentum* 317, *S. thermophilus* 252, *Lb. delbrueckii* 328M, and the yeast *Sc. cerevisiae* strain 370 (mixture A) or the four lactic acid bacteria strains without the yeast (mixture B) (**b**).

The rheological flow curves of the different fermentations carried out are shown in Figure 6. After 24 h fermentation, no significant increase in the viscosity was detected with *nono* fermented with *Lb. delbrueckii* strains 052, 053, and 328M; *L. fermentum* strains 054, 317, and 339; and *Lb. helveticus* strains 058, 314, and 321 (Figure 6a). This was evident as at all shear rates (10,100, and 1000). The *S. thermophilus* strains 252, 363, and 349, on the other hand, were able to thicken the milk and thus the experiment showed typical flow thinning behavior as viscosity decreased with increasing shear rates (Figure 6a, broken lines). The same held true for the co-cultures/mixtures, where viscosity decreased with higher shear rates, which were caused by aggregate destruction (Figure 6b).

### 3.6. Whole Genome Sequence Analysis of Starter Candidates

In order to detect further technological properties from genetic information of the potential starter strains, we sequenced the draft genomes of the selected potential starter bacteria on an Illumina MiSeq. The sequence coverage for the strains ranged from 50.2-fold to 67.1-fold. The sequencing results are shown in Table 1 for the strains *Lb. helveticus* 314 and *S. thermophilus* 252 (this study), while the strains *L. fermentum* strain 317 and *Lb. delbrueckii* 328M were obtained from previous study [23]. In the previous study, we did not analyze the genomes of these strains for technological properties. The starter strains showed genome sizes ranging from 1.78 Mbp for *Lb. delbrueckii* 328M to 2.17 Mbp for *Lb. helveticus* 314, while the mol % GC content was typically low, as is known for LAB [24], ranging from 36.4 to 51.5 (Table 1). The two strains *Lb. helveticus* 314 and *L. fermentum* 317 contained sequences related to plasmid replication protein and an alpha-galactisudase, as well as an alpha-amylase. The *Lb. helveticus* 314 and *S. thermophilus* 252 strains possessed genes for bacteriocin production and self-immunity, while the *Lb. delbrueckii* 328M only possessed a putative bacteriocin immunity gene. All four strains possessed the PTS mannose transporter for sugar uptake, while only *Lb. helveticus* and *Lb. delbrueckii* 328M contained the additional PTS glucitol/sorbitol transporter, showing their greater capacity for sugar utilization.

## 4. Discussion

Generally, the bacterial counts determined on M17 medium incubated at 45 °C in this study were similar to those determined on the same M17 incubated at 30 °C, although in some samples the counts were higher on the latter medium by up to 1 cfu/mL. This indicates that not all the bacteria growing on M17 at 30 °C were streptococci, as the temperature of 45 °C is selective for these bacteria. M17 also supports the growth of lactococci at 30 °C. Similar to our results, Okonkwo [2] reported highly variable counts of different bacterial groups in *nono* samples from Nigerian markets, and in that study, the mesophilic, aerobic plate counts ranged from log 2.53 to log 4.89 per mL, while enterobacterial counts ranged from log 1.37 to log 3.29 per mL. Yeasts were found to be present at levels ranging from log 0.81 to log 1.61 per mL [2]. Overall, our results indicate that lactic acid bacteria (including presumptive streptococci) and yeasts represent predominant microorganisms in *nono* products and that product occasionally also contained Gram-negative bacteria, which are perceived as a hygienic risk. The presence of various pathogenic bacteria in other studies [2,5,6,7,8] confirmed this risk, and this may reflect the fact that *nono* is traditionally produced from non-pasteurized milk under uncontrolled conditions without the use of starter cultures at a household scale.

Metagenomics based on next generation sequencing with an Illumina MiSeq sequencer was performed to investigate the bacterial diversity of *nono* samples. It should be mentioned here that streptococci and lactococci are phylogenetically closely related and that these may not be unequivocally identified on the basis of high throughput sequencing metagenomic studies. This is because only a small portion of the 16S rRNA gene (in this case the V3/V4 region) is sequenced, which may not be sufficiently discriminatory to identify these bacteria to genus level. The varying abundances of LAB in the *nono* products indicate that they were not uniformly fermented and differed regarding the LAB population composition, which exemplifies that the drawback that the milk used in these fermentations was not inoculated with starter bacteria, as the use of an industrial starter would result in a more uniform population if the fermentation process was controlled. It was further noticed that enterobacteria such as *Shigella*, *Escherichia*, and *Klebsiella* occasionally occurred in the fermented products, as was previously reported [2,5,6,7]. It is unlikely that these bacteria will be able to grow and multiply at a pH of 4.0 or less. Nevertheless, the results show that there is an inherent risk of infection by potentially pathogenic bacteria from these products whenever *nono* is not properly fermented. The work of Okonkwo [2] showed that the pH values of *nono* samples from Nigerian markets varied from pH 4.0 to pH 5.4. As suggested by Holzapfel [9], acidification to pH values of less than pH 4.2 constitutes a major safety concern for fermented foods. These data clearly show that there is a need for a controlled fermentation of *nono* type products in order to guarantee their safety.

In order to determine the species composition of the lactic acid bacterial populations more accurately, we identified isolates to the species level by a polyphasic approach based on phenotypic and genotypic methods [14,15]. Repetitive element PCR (rep-PCR) was used as a major fingerprinting method. Overall, the results thus showed that the predominant LAB involved in the *nono* fermentation included *L. fermentum,* together with *Lb. helveticus*, *S. thermophilus*, and *Lb. delbrueckii* strains. The last two microorganisms are well known for their role in yoghurt production and are utilized extensively as starter cultures for the production of this dairy product in western countries. Only few studies have been conducted on the lactic acid bacteria and their role in *nono* fermentation. *L. fermentum,* however, has been often isolated from African fermented foods [25,26,27,28,29,30], possibly as it is associated with vegetable fermentations performed in the same household/production facility. Previously, Okagbue and Bankole [1] also reported *L. fermentum* as the predominant lactic acid bacterium isolated from *nono*. Interestingly, Adekosan et al. and Aforijiku and Onilude [10,29] also isolated *L. fermentum* from *nono*, in addition to other lactic acid bacteria including *Lb. delbrueckii* subsp. *bulgaricus, Lb. plantarum, Lb. brevis*, and *Lb. casei*.

In addition to the lactic acid bacteria, the predominant yeasts isolated from the fermentation in this study were predominantly *Saccharomyces cerevisiae*, as well as, to a lesser extent, *Candida novegensis* strains. This confirms the report of Okagbue and Bankole [1] that *Saccharomyces cerevisiae* can be consistently isolated from *nono* fermentations.

The use of single-strain starter cultures, especially when choosing a predominant *L. fermentum* strain, would probably not lead to a sufficiently deep acidification to ensure the safety of the *nono* product. This could be especially problematic in view of the fact that some potentially pathogenic Gram-negative bacteria were found in these products through the metagenomic microbiota analysis. Thus, for the development of a starter culture for *nono* preparation, it seems necessary to incorporate representative strains of all the predominant lactic acid bacteria species shown to occur in the fermentation. As *Streptococcus thermophilus* strains were noticeably capable of good growth and acid production in the milk, the incorporation of this species appears especially important. Even though *L. fermentum* was revealed as a poor acid producer, it is known, on the other hand, to be involved in aroma production of African fermented foods, as well as for production of extracellular polysaccharides, which may play a role as bio-thickener in the product [27].

Corroborating this position, Okagbue and Bankole [1] also reported the development of starter cultures for *nono* production. The work, however, based the starter cultures for *nono* fermentation on *Lactococcus lactis* subsp. *lactis* biovar diacetylactis (referred to as *Streptococcus diacetilactis* in the original publication), *Lactococcus lactis* subsp. *cremoris* (referred to as *Streptococcus cremoris* in the original publication), *Lb. brevis*, and *Saccharomyces cerevisiae*. Diacetyl production is an important trait influencing the sensory properties and palatability of this product [1]. In their study, *L. lactis* subsp. *lactis* biovar diacetylactis produced *nono* with better taste when compared to *L. lactis* subsp. *cremoris*. Diacetyl production may thus be a key technological property for the starter bacteria used in the production of *nono*. They confirmed that the individual strains showed marked differences in pH-reducing potential and concluded that *Lb. brevis* was a poor acidifier of milk, while the *Lactococcus* strains produced sufficient levels of acidity. The authors concluded therefore that single microorganism starter cultures should be avoided and that *L. lactis* could be used together with *Lb. brevis* as starter culture. As similar results were obtained also in our study, various starter cultures containing one representative strain of each of the four predominant LAB species were tested to determine their effect on functional aspects of *nono* production, including milk acidification and rheological properties. Similar to Okagbue and Bankole [1], we also opted to test these starter cultures either with or without addition of the yeast *Saccharomyces cerevisiae*.

Both starter combinations tested achieved a pH reduction from 6.5 to less than 4.0, a pH level that according to Holzapfel [9] would suffice in terms of guaranteeing the safety of these fermented products. As seen from single-strain incubations, this was probably the result of the high acid-producing capacity of the *S. thermophilus* strain 252. These results indicate that the use of starter culture strains could result in products with high levels of these bacteria that stabilized the products by acidification to a pH of 4.0. Furthermore, varying levels of microorganisms of different bacterial groups, as determined in products obtained on Nigerian markets, could be avoided by the use of starter cultures, which would quickly dominate the fermentation. We did not examine yeast as single starter culture. Hence, whether or not *Saccharomyces cerevisiae* plays an important role in the fermentation has still to be determined in more detail. Previously, Okagbule and Bankole [1] had suggested that the incorporation of the yeast did not result in better sensory characteristics or higher diacetyl levels, and concluded that it was relatively unimportant in *nono* production and that its use in a commercial situation would be unnecessary.

Data from the rheological study corresponds well with the results of the pH measurements, which showed that *Lb. delbrueckii*, *L. fermentum*, and *Lb. helveticus* strains from *nono* did not lower the pH below 5.0 and were thus not able to gel the milk. Again, the flow thinning behavior of *S. thermophilus* could be explained by the higher acid-producing capacity of the *S. thermophilus* strains, which could lower the pH of the milk during fermentation to below pH 5.0, causing the milk to gel and to thicken.

The *Lb. helveticus* 314 and *L. fermentum* 317 strains possessed the gene for citrate lyase [23], which can be important for citrate metabolism and for the potential production of the aroma compound diacetyl. The genomes of strain *L. fermentum* 317 and *S. thermophilus* 252 also contained genes for an acetolactate synthase that may also be involved in diacetyl production. Only the genome of *Lb. delbrueckii* 328M did not show the presence of any genes that may be related to diacetyl production. Diacetyl production has previously been described to be important for starter bacteria involved in *nono* production [1], and thus the starters selected in this study appeared to possess the genes required for this important technological function. The presence of the signature genes for folate biosynthesis (dihydropteroate synthase (*folP*) and 2-amino-4-hydroxy-6-hydroxymethyldihydropteridine diphosphokinase (*folK*)) indicates that the starter (culture) strains may hypothetically be capable of folate production. This is another functionally important trait for starter bacteria for African fermented foods [31]. The *Lb. helveticus* 314 and *S. thermophilus* 252 strains possessed the bacteriocin genes for a class III bacteriocin and class IIb bacteriocin, respectively. The production of bacteriocin may hypothetically be helpful for inhibiting Gram-positive pathogenic bacteria, and thus these starter cultures may contribute to the safety of *nono* products.

## 5. Conclusions

Overall, our metagenomic and, more importantly, strain typing study showed that lactobacilli and streptococci dominated the lactic acid microbiota of fermented *nono.* The bacterial species predominantly determined to occur were *Lb. helveticus*, *L. fermentum*, *Lb. delbrueckii*, and *S. thermophilus* strains, while the predominant yeast occurring in *nono* was *Saccharomyces cerevisiae*. Starter cultures development for *nono* production should thus be based on a combination of these predominant strains. *Lb. delbrueckii* and *S. thermophilus* are typical yoghurt starter bacteria that grow synergistically. Using metagenomics, pathogens such as *Shigella* and potential pathogens such as enterobacteria could be detected at low abundances. Starter selection for *nono* fermentation should include suitable technological strains of the bacterial species that are predominantly occurring in *nono*, i.e., *Lb. helveticus*, *L. fermentum*, *Lb. delbrueckii*, and *S. thermophilus* strains. However, the predominant *L. fermentum*, *Lb. helveticus*, and *Lb. bulgaricus* strains did not show a high capacity for acid production and consequently for milk gelling as a result of pH decrease. *S. thermophilus* strains, on the other hand, showed good acidification properties, and rheological results showed the strains’ capacity for gelling of milk to a yogurt consistency. As *Lb. delbrueckii* and *S. thermophilus* are known to support each other’s growth in yoghurt, *Lb. delbrueckii* was considered an important starter also for the production of *nono*. *L. fermentum* and *Lb. helveticus* may, on the other hand, also be important for production of flavor metabolites such as diacetyl. In this study, therefore, predominant strains were selected for further development as starter bacteria. These strains show that when used in combination as starters, they are effective in growing and lowering the pH of milk, causing gelling of the product to a yoghurt-like consistency. All strains, except for the *Lb. delbrueckii* strain, possess genes potentially involved in diacetyl production and folate biosynthesis signature genes. These strains should therefore be further studied in Nigeria to test for their success in *nono* fermentations under local manufacturing conditions.

## Figures and Tables

**Table 1:** Results of genome sequencing of representative predominant strains of *nono* production. The genome sequence data for *L. fermentum* 317 and *Lb. delbrueckii* 328M were obtained previous study [23]. +; detected, −; not detected

Strain	*Lb. helveticus* 314	*L. fermentum* 317	*Lb. delbrueckii* 328M	*S. thermophilus* 252
No. of contigs	132	144	73	74
Largest contig	156,761	133,516	135,635	180,564
N50	29,559	43,204	60,623	59,820
GC content (mol%)	36.46	51.55	49.72	38.67
Total length (bp)	2,169,532 bp	1,924,745 bp	1,785,290 bp	1,814,605 bp
Plasmid sequence	+	+	−	−
CDS (coding sequence)	2270	1950	1815	1844
tRNA	60	54	74	41
rRNA	4	10	9	4
Bacteriocin	+	−	−	+
Citrate lyase	+	+	−	−
Acetolactate synthase	−	+	−	+
Dihydropteroate synthase	+	+	+	+
2-Amino-4-hydroxy-6-hydroxymethyldihydropteridine diphosphokinase	−	+	−	+
Alpha-galactosidase	+	+	−	−
Alpha-amylase	+	+	−	−
PTS mannose/fructose/sorbose transporter	+	+	+	+
PTS glucitol/sorbitol transporter	+	−	+	−

## Data Availability

All genome data generated in this study was deposited in the National Center for Biotechnology Information public sequence database under BioProject ID PRNJA578299.

## Appendix A

**Table A1:** Yeast identification based on sugar utilization as tested using the API ID 32C, showing the API identification code that resulted in the strain identification at the apiweb (BioMérieux) database.

Species	Isolate	API	GAL	ACT	SAC	NAG	LAT	ARA	CEL	RAF	MAL	TRE	2KG	MDG	SOR	XYL	RIB	GLY	RHA	PLE	ERY	MEL	GRT	MLZ	GNT	LVT	MAN	LAC	INO	GLU	SBE	GLN	ESC
*Saccharomyces cerevisiae*	103	5260000001	+	−	+	−	+	−	−	+	+	−	−	−	−	−	−	−	−	−	−	−	−	−	−	−	−	−	−	+	−	−	−
	104	5260000011	+	−	+	−	+	−	−	+	+	−	−	−	−	−	−	−	−	−	−	−	−	−	−	−	+	−	−	+	−	−	−
	106	5200000001	+	−	+	−	+	−	−	−	−	−	−	−	−	−	−	−	−	−	−	−	−	−	−	−	−	−	−	+	−	−	−
	109	5320400001	+	−	+	−	−	−	−	+	−	−	−	−	−	−	+	−	−	−	−	−	−	−	−	−	−	−	−	+	−	−	−
	111	1244400001	+	−	+	+	+	−	−	+	+	−	−	+	−	−	+	−	−	−	−	−	−	−	−	−	−	−	−	+	−	−	−
	113	5470000001	+	−	+	−	+	−	+	+	+	−	−	−	−	−	−	−	−	−	−	−	−	−	−	−	−	−	−	+	−	−	−
	115	1240000001	+	−	−	−	+	−	−	−	+	−	−	−	−	−	−	−	−	−	−	−	−	−	−	−	−	−	−	+	−	−	−
	191	5060000001	+	−	+	−	−	−	−	−	−	−	−	−	−	−	−	−	−	−	−	−	−	−	−	−	−	−	−	+	−	−	−
	193	5060000001	+	−	+	−	−	−	−	−	−	−	−	−	−	−	−	−	−	−	−	−	−	−	−	−	−	−	−	+	−	−	−
	202	5060000001	+	−	+	−	−	−	−	−	−	−	−	−	−	−	−	−	−	−	−	−	−	−	−	−	−	−	−	+	−	−	−
	206	5040000001	+	−	+	−	−	−	−	−	+	−	−	−	−	−	−	−	−	−	−	−	−	−	−	−	−	−	−	+	−	−	−
	370	5220000001	+	−	+	−	−	−	−	+	+	−	−	−	−	−	−	−	−	−	−	−	−	−	−	−	−	−	−	+	−	−	−
	371	5060000001	+	−	+	−	−	−	−	−	+	−	−	−	−	−	−	−	−	−	−	−	−	−	−	−	−	−	−	+	−	−	−
	372	5220000001	+	−	+	−	+	−	−	+	−	−	−	−	−	−	−	−	−	−	−	−	−	−	−	−	−	−	−	+	−	−	−
	373	5260000001	+	−	+	−	+	−		+	+	−	−	−	−	−	−	−	−	−	−	−	−	−	−	−	−	−	−	+	−	−	−
	377	5260000001	+	−	+	−	+	−	−	+	+	−	−	−	−	−	−	−	−	−	−	−	−	−	−	−	−	−	−	+	−	−	−
	378	5260000001	+	−	+	−	+	−	−	+	+	−	−	−	−	−	−	−	−	−	−	−	−	−	−	−	−	−	−	+	−	−	−
*Pichia norvegensis*	105	O200000001	−	−	−	−	+	−	−	−	−	−	−	−	−	−	−	−	−	−	−	−	−	−	−	−	−	−	−	+	−	−	+
	114	O200010001	−	−	−	−	+	−	−	−	−	−	−	−	−	−	−	+	−	−	−	−	−	−	−	−	−	−	−	+	−	−	−
	116	O200010001	−	−	−	−	+	−	−	−	−	−	−	−	−	−	−	+	−	−	−	−	−	−	−	−	−	−	−	+	−	−	+
	190	O200010001	−	−	−	−	+	−	−	−	−	−	−	−	−	−	−	+	−	−	−	−	−	−	−	−	−	−	−	+	−	−	−
	192	O200010001	−	−	−	−	+	−	−	−	−	−	−	−	−	−	−	−	−	−	−	−	−	−	−	−	−	−	−	+	−	−	−
	199	O200000001	−	−	−	−	+	−	−	−	−	−	−	−	−	−	−	−	−	−	−	−	−	−	−	−	−	−	−	+	−	−	−
	200	O200010001	−	−	−	−	+	−	−	+	−	−	−	−	−	−	−	−	−	−	−	−	−	−	−	−	−	−	−	+	−	−	−
	369	O200000001	−	−	−	−	+	−	−	−	−	−	−	−	−	−	−	−	−	−	−	−	−	−	−	−	−	−	−	+	−	−	−
	375	O200000001	−	−	−	−	+	−	−	−	−	−	−	−	−	−	−	−	−	−	−	−	−	−	−	−	−	−	−	+	−	−	−
*Kluyveromyces marxianus*	101	7200300021	+	+	+	−	+	−	−	−	−	−	−	−	+	+	−	−	−	−	−	−	−	−	−	−	−	+	−	+	−	−	+
	108	7220200021	+	+	+	−	+	−	−	−	−	−	−	−	+	−	−	−	−	−	−	−	−	−	−	−	−	+	−	+	−	−	+
	374	7220300021	+	+	+	−	+	−	−	+	−	−	−	−	+	+	−	−	−	−	−	−	−	−	−	−	−	+	−	+	−	−	+
*Pichia kudriavzevii*	110	1300010003	+	−	−	+	+	−	−	−	−	−	−	−	−	−	−	+	−	−	−	−	−	−	−	−	−	−	−	+	+	−	+
	112	O300010001	−	−	−	+	+	−	−	−	−	−	−	−	−	−	−	+	−	−	−	−	−	−	−	−	−	−		+	−	−	+
	367	O300010001	−	−	−	+	+	−	−	−	−	−	−	−	−	−	−	+	−	−	−	−	−	−	−	−	−	−		+	−	−	+
*Kasachstania holmii*	107	5020000001	+	−	+	−	−	−	−	+	−	−	−	−	−	−	−	−	−	−	−	−	−	−	−	−	−	−	−	+	−	−	−
	201	5020000001	+	−	+	−	−	−	−	+	−	−	−	−	−	−	−	−	−	−	−	−	−	−	−	−	−	−	−	+	−	−	−
*Candida sake*	368	5143350113	+	−	+	+	−	−	−	−	+	+	+	−	+	+	−	+	−	+	−	−	−	+	−	−	−	−	−	+	−	−	−
*Clavispora lusitaniae*	198	5157170307	+	−	+	+	−	−	+	−	+	+	+	+	+	−	−	+	+	+	−	−	−	+	+	−	−	−	−	+	+	+	+
*Diutina rugosa*	102	1300300011	+	−	−	+	+	−	−	−	−	−	−	−	+	+	−	−	−	−	−	−	−	−	−	−	+	−	−	+	−	−	−
*Geotrichum ssp.*	195	3000310001	+	+	−	−	−	−	−	−	−	−	−	−	+	−	−	−	−	−	−	−	−	−	−	−	−	−	−	+	−	−	−
*Kasachstania ichnusensis*	376	5020000001	+	−	+	−	−	−	−	+	−	−	−	−	−	−	−	−	−	−	−	−	−	−	−	−	−	−	−	+	−	−	−
*Naganishia albida*	197	4457364141	−	−	+	−	−	+	+	−	+	+	+	+	+	+	−	−	+	+	−	−	+	+	−	−	+	−	−	+	+	−	−
*Pichia membranaefaciens*	210	O100010001	−	−		+	−	−	−	−	−	−	−	−	−	−	−	+	−	−	−	−	−	−	−	−	−	−	−	+	−	−	−
*Rhodotorula mucilaginosa*	196	5461740111	+	−	+	−	−	+	−	+	+	+	−	−	+	+	+	−	−	−	−	−	−	+	−	−	+	−	−	+	−	−	−
*Zygosaccharomyces ssp.*	205	1000000001	+	−	−	−	−	−	−	−	−	−	−	−	−	−	−	−	−	−	−	−	−	−	−	−	−	−	−	+	−	−	−

GAL, D-GALactose; ACT, cycloheximide(ACTidion); SAC, D-SACcharose (sucrose); NAG, N-Acetyl-Glucosamine; LAT, LAcTic acid; ARA, L-ARAbinose; CEL, D-CELobiose; RAF, D-RAFfinose; MAL, D-MALtose; TRE, D-TREhalose; 2KG potassium-2-KetoGluconate; MDG, Methyl-aD-Glucopyranoside; MAN, D-MANnitol; LAC, D-LACtose (bovine origin); INO, INOsitol; SOR, D-SORbitol; XYL, D-XYLose; RIB, D-RIBose; GLY, GLYcerol; RHA, L-RHAmnose; PLE, PaLatinosE; ERY, ERYthritol; MEL, D-MELibiose; GRT, sodium GlucuRonaTe; MLZ, D-MeLeZitose; GNT, potassium; GlucuNaTe; LVT, levulinic acid (LeVilinaTe); GLU, D-GLUcose; SBE L-SorBosE; GLN, GLucosamiNe; ESC, ESCulin ferric citrate. +; detected, −; not detected.
